# Ethylenediurea reduces grain chalkiness in hybrid rice cultivars under ambient levels of surface ozone in China

**DOI:** 10.3389/fpls.2022.983576

**Published:** 2022-09-01

**Authors:** Guoyou Zhang, Hamdulla Risalat, Kazuhiko Kobayashi, Rong Cao, Qinan Hu, Xiaoya Pan, Yaxin Hu, Bo Shang, Hengchao Wu, Zujian Zhang, Zhaozhong Feng

**Affiliations:** ^1^Key Laboratory of Agrometeorology of Jiangsu Province, School of Applied Meteorology, Nanjing University of Information Science and Technology, Nanjing, China; ^2^Jiangsu Key Laboratory of Crop Genetics and Physiology, Agricultural College of Yangzhou University, Yangzhou, China; ^3^The University of Tokyo, Tokyo, Japan; ^4^Chang Wang School of Honors, Nanjing University of Information Science and Technology, Nanjing, China; ^5^College of Wetland, Southwest Forestry University, Kunming, China; ^6^Agricultural College, Yangzhou University, Yangzhou, China

**Keywords:** environmental pollution, climate change, food security, appearance quality, ozone indicator, ozone protectant, rice type, chalkiness type

## Abstract

High concentration of tropospheric ozone (O_3_) causes crop yield losses, which could be reduced by foliar application of ethylenediurea (EDU). Rice grain appearance is a major quality trait that determines the milling quality, white rice productivity and the market value. Grain chalkiness is one of the common defects that deteriorate the grain appearance in rice due to its negative effects on palatability and milling yield. Whether EDU could reduce grain chalkiness in rice which was usually increased by high concentration of O_3_ is not clarified. We report the grain chalkiness in 19 rice cultivars (CVs) of three types: indica (6 CVs), japonica (5 CVs) and hybrids (8 CVs), observed in an EDU application experiment in the field in China. The ambient O_3_ level as expressed by accumulated hourly O_3_ concentration over the threshold of 40 ppb (AOT40) for 80 days until maturity reached 12.8 ppm h at a near-by monitoring station. Fraction of the chalky grains (FCG) in the hybrid cultivars was 8% lower in EDU than that in the control treatments, whereas no significant effect of EDU on FCG was found in japonica or indica cultivars. The reduction of FCG due to EDU treatment in hybrid cultivars was attributed to the significant reduction of milky white grains followed by that of white belly grains. Thus, the application of EDU could ameliorate the decline of grain appearance quality in hybrid rice by decreasing the FCG and enhancing the fraction of perfect grains (FPG). Moreover, there were significant interactions between the EDU application and rice cultivars, indicating varietal difference in the protection of grain appearance quality by EDU. These results suggest the need for further studies on the mechanisms of the effects of EDU on grain chalkiness.

## Introduction

Ground-level ozone (O_3_) concentration in Asia has been rising particularly at an alarming rate in recent years (Lu et al., 2020) in contrast to its declining trends in Europe and most part of North America (Mills et al., 2018). The rise of O_3_ level in Asia poses threats to production of various crop species (Feng et al., 2020). Since rice (*Oryza sativa* L.) is the most important crop as a staple food for the majority of population and the major income source for many small-holder farmers (Muthayya et al., 2014), the impacts of rising levels of O_3_ on rice is a major threat to food supply and local livelihood in Asia.

Many studies have shown the reduction of grain yield of rice in experiments with elevated levels of O_3_ (Wang et al., 2012; Shao et al., 2020). Using the results of the experiments, O_3_-induced harvest losses of rice have been estimated at various scales from a region to the entire globe (Feng et al., 2022). In comparison to the concern on the loss of harvested amount of rice grains, however, less attention has been paid to the deterioration of rice grain quality under elevated O_3_ levels (Wang et al., 2012).

Since rice is sold and consumed in grains, the grain quality is a determinant of food security and household economy (Busanello et al., 2020). Among the various quality traits, grain chalkiness is of major concern because it deteriorates milling yield and palatability of cooked rice (Kim et al., 2000; Chun et al., 2009) resulting in lower grading and price of the chalky grains on the market. This is why the incidences of increased grain chalkiness under rising air temperature have become a major issue for rice production (Kim et al., 2000; Chun et al., 2009; Cheng et al., 2019). It is also reported that elevated concentrations of atmospheric CO_2_ increased the incidence of chalky rice grains (Usui et al., 2016). The prospect of more incidences of chalky grains under rising air temperature and CO_2_ levels is only exacerbated by the reported increase of chalky grains in rice plants under elevated O_3_ concentrations (Wang et al., 2012; Jing et al., 2016; Sawada et al., 2016).

To quantify the risk of increased chalky grains due to surface O_3_, however, the past studies have been too much limited in their coverage of genetic variability. They investigated a hybrid cultivar (Wang et al., 2012), compared between a japonica and an indica inbred cultivars (Sawada et al., 2016), or compared one japonica inbred cultivar with the japonica-based introgression line (Jing et al., 2016). No direct comparison has been made among more than two cultivars across the rice types of indica, japonica and hybrid on grain chalkiness under elevated O_3_. Of a particular concern is the comparison between hybrid and inbred type cultivars. Both types of cultivars have exhibited the increased grain chalkiness due to elevated O_3_ levels but in separate studies, which differed in the O_3_ levels as well as the other experimental conditions. Since the grain chalkiness is caused by anomaly in starch accumulation in the rice grains (Ishimaru et al., 2009; Lo et al., 2016), the greater decline of the flag leaf photosynthesis in hybrid than inbred cultivars under elevated O_3_ concentration could lead to higher incidences of chalky grains in hybrids. Greater incidences, if any, of the anomalous grain quality would have vast impacts on rice production in China, where hybrid type cultivars are quite popular for their higher yields than the inbred ones (Hu et al., 2016).

In the past studies that reported increased grain chalkiness, the experiments have been conducted in O_3_ elevation facilities, where the available area for the plant growth was limited and so was the number of genotypes investigated. The FACE (free-air ozone concentration elevation) apparatus could be scaled up to accommodate more cultivars, which, however, would demand more technological and financial resources. An alternative to FACE for screening multiple cultivars under O_3_ stress is to apply an antiozonant chemical such as ethylenediurea (EDU) to the crop plants. EDU can be applied to the plants in the field *via* foliar spraying or soil drench just like any other agrochemicals. EDU application has indeed been adopted to assess the phytotoxicity of O_3_ and to screen tolerant cultivars in various crop species (Paoletti et al., 2009; Manning et al., 2011; Oksanen et al., 2013; Ashrafuzzaman et al., 2017).

We therefore adopted EDU in this study for application to rice plants of 19 cultivars chosen from the three types: indica inbred, japonica inbred and hybrid, and compared occurrence of chalky grains among the rice types and cultivars. This is actually an additional observation of the grains harvested in an EDU application experiment to quantify the O_3_ impacts on grain yield and yield components as reported elsewhere (Zhang et al., 2021), but is notably the first ever investigation of changes in rice grain appearance by EDU application.

In this study, we hypothesized that the grain chalkiness shall be reduced by EDU application *via* the protection from damages by moderately high levels of O_3_ in ambient air at the site of experiment (Zhang et al., 2021). On the comparison between hybrid and inbred types, however, we had two contrasting hypotheses. Hybrid cultivars have exhibited greater responses in plant growth and yield to elevated O_3_ than inbred ones (Wang et al., 2012), which points to a greater response of grain chalkiness to EDU application. On the other hand, our EDU application experiment showed no difference between the rice types in the increase of rice yield and yield components (Zhang et al., 2021), which suggests the reduction of grain chalkiness by EDU to the same extent across the rice types. We also hypothesized that there is a difference between cultivars within a rice type, as was the case in the comparisons of grain chalkiness under elevated O_3_ between inbred varieties (Sawada et al., 2016; Jing et al., 2016). We investigated the grain appearance traits and compared them among the cultivars and rice types to test the above hypotheses.

## Materials and methods

### Experimental site

The experiment was conducted in a paddy field (32°16′N, 119°33′E) located in Zhenjiang City, Jiangsu Province, China in 2018. The site of experiment is in a lower reach of the Yangtze River Delta (YRD), which is one of the major rice production areas in China but is under rising levels of surface O_3_. In the EDU application experiment, we have estimated that AOT40 (daytime-hours surface O_3_ concentrations above the threshold of 40 ppb) at 12.8 ppm h for an accumulation period of 80 days until maturity (Zhang et al., 2021). This AOT40 value in the ambient air is closer to the AOT40 value (15.5 ppm h) in the elevated O_3_ plots than the ambient O_3_ plots (7.7 ppm h) recorded in an O_3_ elevation experiment conducted at another field in the same province (32°35′N, 119°42′E) in 2007. Since the latter AOT40 values are on a 90 days period of accumulation, the AOT40 value accumulated for 80 days in this study would come closer to that in the elevated O_3_ plots in 11 years before.

### Crop cultivation and ethylenediurea application

In a paddy field of 46 × 35 m, we set 10 plots (9.6 × 5.5 m each), of which 5 plots were assigned to the EDU treatment and the other 5 plots were for the Control treatment. In each plot, we investigated 19 cultivars, of which 8 cultivars were hybrid, 6 were indica inbred, and 5 were japonica inbred (Supplementary Figure 1). Seeds of rice cultivars were sown in the nursery in mid-May of 2018, and the seedlings were planted in the field with a density of 28 hills m^–2^ (one seedling per hill) on 16 June. Fertilizer and water management, pest and animal controls as well as other field practices were performed following the local farmers’ practices.

Ethylenediurea solution (450 ppm) was prepared by dissolving EDU powder (100%) in warm water, in order to increase solubility, 0.01% of Tween 20 was added (Yuan et al., 2015; Feng et al., 2018; Jiang et al., 2018; Zhang et al., 2021). This concentration appears to be the maximum level in the low-dose range applied as a protectant against O_3_, and indeed effectively protected a hybrid rice cultivar from yield loss due to ambient level of O_3_ (Shang et al., 2022). We conducted the first foliar spray of plants with the EDU solution from 8:00 to 9:00 AM on 26 June, and repeated the spraying for 9 more times at an interval of 10 days. At the same time of the EDU spraying, we sprayed the plants in Control treatment with the same volume of Tween 20 buffer solution without EDU. More details of the EDU application have been given in Zhang et al. (2021).

### Plant sampling and grain appearance analysis

Each cultivar was harvested at physiological maturity, when 85% of the grains became straw-colored and the grain moisture content fell to ca. 20% (Zhang et al., 2021). All grains per plant were airdried to constant weight. After determining the grain yield, sub samples were collected for the grain quality analysis. We took 100 dehulled grains from each subplots, and visually inspected the individual grains for chalkiness and other anomalies. We classified the chalky grains into five types according to the position of the chalky appearance within a rice kernel: grains with white-back (WBcG), white-base (WBsG), white-belly (WBlG), white-core (WCG), and milky-white (MWG) in reference to Figure 1 of Yoshioka et al. (2007). We then aggregated them together as chalky grains (ACG). The grains with good shape and no anomalies were counted as undamaged grains (UDG). We then calculated fractions of grains in the above categories in 100 grains as a means for quantifying the effects of EDU application on grain appearance quality. More details for the visual inspection are described in Supplementary material.

**FIGURE 1:**
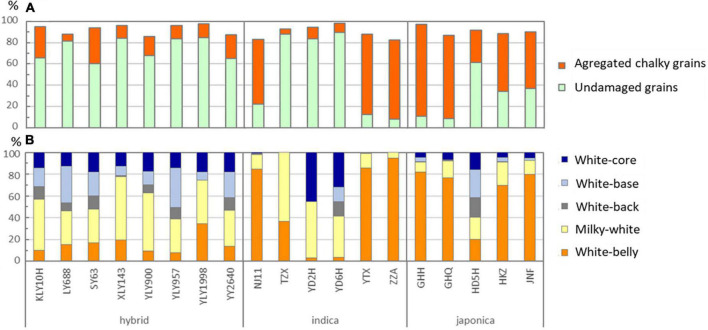
Fractions of undamaged grains and aggregated chalky grains **(A)**, and fractions of various chalkiness types in the aggregated chalky grains **(B)** by cultivar and rice type on average across the EDU and Control treatment levels.

The results of grain appearance analysis were subjected to ANOVA by fitting a mixed-linear model based on the random effect of the field plot and the fixed effects of rice type (indica, japonica, or hybrid), EDU application (EDU or Control), interaction between rice type and EDU application, cultivar nested in rice type, and interaction between cultivar and EDU application. Fractions of chalky grains of any types were subjected to the logit transformation where necessary to correct for heterogeneity of the error variance. Fractions of various types of chalky grains and perfect grains were compared between the EDU and Control treatments with the *t*-test separately for each of the three rice types, while the overall Type-I error across the 3 comparisons was controlled by applying the Bonferroni correction. In the same way, fractions of various types of grain chalkiness were compared among the three rice types. Comparison of grain appearance trait between EDU and Control treatments was also done with the *t*-test for each of the 19 cultivars, and the overall Type-I error across the 19 comparisons was controlled by applying the Bonferroni correction. The statistical analysis was done with JMP-Pro ver. 16 (SAS Institute, Cary, United States).

## Results

### Genetic variability in the grain appearance traits

When averaged across the EDU treatments, fraction of the perfect grains was reduced by the increased fraction of chalky grains to the extent varied by the rice type and cultivars (Figure lA). The effects of rice type and cultivar on the fractions of undamaged grains (UDG) and aggregated chalky grains (ACG) were indeed highly significant (Table 1). The fraction of UDG was highest in hybrid followed by indica and japonica types in this order, whereas the opposite was true in the fraction of ACG (Figure 1A and Table 1). The difference in ACG fraction between the rice types was mostly attributed to the higher fractions of white-belly grains (WBlG) in japonica followed by indica types as compared with that in hybrid type (Table 1). The WBlG fraction accounted for the largest fraction of ACG in japonica and indica types but was a minor component in hybrid type when averaged across cultivars (Table 1). In each rice type, however, the contribution of WBlG to ACG varied greatly among the cultivars (Figure 1B).

**TABLE 1:** Effects of ethylenediurea (EDU) application on the grain appearance traits (UDG, undamaged grains; ACG, aggregated chalky grains; WBlG, white-belly grains; MWG, milky-white grains; OtCG, other types of chalky grains consisting of white-back, white-base and white-core grains) in interaction with the rice type and cultivar.

Effect	*P*-value for the effect on the fraction of grains in the appearance category				
	UDG	ACG	Type of grain chalkiness		
			WBlG^a^	MWG	OtCG^a^
EDU treatment	0.949	0.540	0.112	0.751	0.383
Rice type	**<0.001**	**<0.001**	**<0.001**	**0.004**	**<0.001**
Rice type x EDU	**<0.001**	**<0.001**	**0.007**	**0.002**	**<0.001**
Cultivar	**<0.001**	**<0.001**	**<0.001**	**<0.001**	**0.001**
Cultivar x EDU	0.143	0.323	0.234	0.098	**<0.001**
**Rice type**	**LSM (%) ^b^ of the fraction of grains in the appearance category by rice type**				
Hybrid	74.1 a	18.4 a	2.61 a	7.47 ab	7.07 a
Indica	50.6 b	39.1 b	11.0 b	5.76 a	1.27 b
Japonica	30.4 c	60.4 c	39.5 c	8.94 b	6.83 a

a. Fractions of WBlG and OtCG were subjected to the logit-transformation for stabilizing the error variance which was larger for higher fractions.

b. Least-square means sharing the same letter are not statistically different within each appearance category, where the Type-I error across the 3 comparisons (hybrid-indica, hybrid-japonica, indica-japonica) is controlled at 0.05 with the Bonferroni correction.

Significance of the effects is shown with the p-value, and comparison of the effects among the rice types is presented by the least-square means (LSM). Bold indicates the significant values (P < 0.05).

Fraction of the milky-white grains (MWG) differed less than that of WBlG between the rice types, of which hybrid type did not significantly differ from either of the inbred types (Table 1). The large relative contribution of MWG to ACG in hybrid type (Figure 1B) can therefore be attributed to the lower occurrence of WBlG in hybrid rather than an increase of MWG. The other 3 types of grain chalkiness: grains with white-back (WBcG), white-base (WBsG) and white-core (WCG), were aggregated to other types of chalky grains (OtCG), since their fractions were individually minor in many cultivars and the 3 types of grain chalkiness often occurred concomitantly (Figure 1B). Indica type showed significantly less fraction of OtCG than japonica and hybrid types (Table 1).

### Effects of ethylenediurea application on the grain appearance traits

Effects of EDU application on fractions of UDG as well as ACG differed between the rice types as indicated by the highly significant interaction between EDU application and rice type (Table 1). Only hybrid type showed significant effects of EDU on the grain appearance traits. Across the hybrid type cultivars, EDU application increased the fraction of UDG from 70 to 78%, whereas it reduced the fraction of ACG from 22 to 14% (Table 2). It was reduced fractions of MWG and WBlG that resulted in the reduced ACG due to EDU application. The fraction of OtCG, which is the aggregate of white-back, white-base and white-core grains, showed no significant effect of EDU application in hybrid type but was significantly increased by EDU in japonica type (Table 2).

**TABLE 2:** Effects of EDU application on grain appearance traits in each rice type as shown by the least-square means (LSM) and *p*-values.

Rice type	EDU treatment	UDG		ACG		Type of grain chalkiness					
						WBlG^a^		MWG		OtCG^a^	
						
		LSM (%)	p^b^	LSM (%)	p^b^	LSM (%)	p^b^	LSM (%)	p^b^	LSM (%)	p^b^
Hybrid	Control	70.0	**<0.001** **	22.4	<0.001**	3.47	0.003**	9.0	0.004 *	8.31	0.043
	EDU	78.2		14.5		1.95		5.9		6.01	
Indica	Control	53.3	0.025	37.2	0.082	10.2	0.364	6.1	0.588	1.15	0.248
	EDU	47.9		41.0		11.9		5.4		1.41	
Japonica	Control	31.7	0.308	59.3	0.339	42.8	0.193	7.4	0.018	5.46	0.014 *
	EDU	29.1		61.5		36.3		10.5		8.51	

a. Fractions of WBlG and OtCG were subjected to the logit-transformation for stabilizing the error variance which was larger for higher fractions of the respective type. LSM calculated for these traits were transformed back to the original scale (%).

b. The p-value is for the t-test of the Control-EDU contrast in each type of rice. Those shown in bold letters with asterisks are deemed statistically significant with the Type-I error across the 3 contrasts controlled at 0.05 (*) and 0.01 (**) after the Bonferroni correction (multiplication of the p-value by 3).

While there was highly significant effect of cultivars on all the chalkiness types, the interaction between EDU and cultivar was not significant in all the grain appearance traits but OtCG (Table 1). The above-noted increase of UDG and decrease of ACG in hybrid type due to EDU application can therefore be viewed as a common response among the cultivars.

**FIGURE 2:**
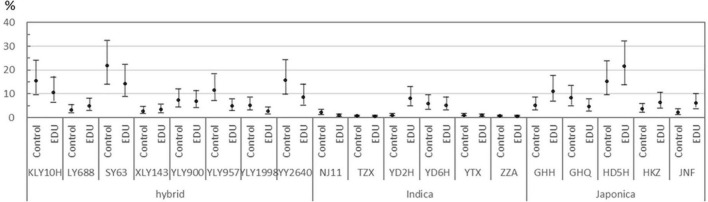
Fraction of OtCG (other types of chalky grains): an aggregate of white-back, white-base, and white-core grains, for each cultivar of hybrid, indica, and japonica types The least square mean (filled circle) and its 95% confidence interval (short horizontal lines connected with a vertical line) are shown for comparison between EDU and Control treatments. Note that the least-square means and confidence intervals were estimated with the logit-transformed fraction of OtCG, but presented in the original scale (%) herein, which is why the confidence interval is non-uniform across the scale of OtCG fraction.

Only the fraction of OtCG, an aggregate of white-back, white-base, and white-core grains, showed significant effect of cultivar by EDU interaction (Table 1), which is investigated by comparing the least-square mean (LSM) and its 95% confidence interval between EDU and Control treatments for individual cultivars (Figure 2). Most cultivars of hybrid type showed lower LSM in EDU than Control, but the overlapping confidence intervals between EDU treatments suggested no significant difference. In japonica type also, the confidence intervals overlapped between EDU treatments suggesting no significant difference, although the LSM appeared to be higher in EDU than Control for 4 out of 5 japonica cultivars (Figure 2). Among the 19 cultivars, only the indica cultivar YD2H showed clear separation of confidence intervals between EDU and Control treatments on this category of grain chalkiness (Figure 2), which was in fact the white-core grains (Figure 1B). This judgment based on the comparison of confidence intervals was supported by the *t*-test of the EDU-Control contrast for each cultivar. Effect of EDU application on the fraction of OtCG in cultivar YD2H was statistically significant with the Type-I error controlled at 0.01 across the 19 comparisons by applying the Bonferroni correction. None of the other 18 cultivars showed significant effect of EDU application with the Type-I error controlled at 0.10 across all the 19 comparisons.

## Discussion

The results of our experiment support the hypothesis that EDU reduces chalky grains (ACG) and thereby increases undamaged grains (UDG) in hybrid type cultivars. It is therefore suggested that the grain appearance quality in hybrid cultivars is deteriorated by surface O_3_ at the current levels in the major rice producing area of this study.

The lack of significant effects of interaction between cultivar and EDU on ACG and UDG supports the assumption that the current levels of O_3_ increases the grain chalkiness across the hybrid cultivars. Considering the wide use of hybrid cultivars in China (Hu et al., 2016) and fast increase of surface O_3_ concentrations in most parts of the country (Lu et al., 2020), we need to quantify the economic impacts of the deteriorating grain quality in addition to those of the reduced amount of the grain harvest (Feng et al., 2022). It must also be recognized that EDU application may not completely protect the rice plants particularly of hybrid cultivars from negative impacts of O_3_. This argument was made on the grain yield response to EDU application with the same hybrid cultivars in the experiment from which we took the grain samples for this study (Zhang et al., 2021). Since no study has ever been done on grain quality with a combination of EDU and O_3_ elevation treatments, we cannot quantify the efficiency of protection by EDU from the increased grain chalkiness due to O_3_. The changes in the grain appearance traits by EDU application in this study may therefore only underrepresent the true O_3_ impacts.

It is interesting to note that different types of grain chalkiness responded differently to EDU. Milky white and white belly was reduced by EDU in hybrid cultivars. However, in inbred, the other chalkiness types were increased across japonica cultivars and in an indica cultivar (YD2H). Mechanisms of the increased chalkiness in the inbred cultivars due to EDU could possibly be related with that the EDU application increased the panicle density, which means that the plants had more sinks under EDU than control, and increased sink size is more conducive to grain chalkiness (Kobata et al., 2004). Different response of grain growth in rice cultivars to EDU could have also induced the variations in the changes of grain chalkiness in different cultivars, as our previous studies have reported that EDU did not extend the period of grain filling in inbred cultivars but extended it in hybrid cultivars (Zhang et al., 2021). Grain filling, by which the seed growth was completed, could also induce the occurrence of grain chalkiness through allocation of carbohydrate and other nutrition. Jing et al. (2016) reported that high concentration of surface ozone (100 ppb) significantly increased grain chalkiness in a sensitive cultivar, through reduced photosynthate supply during the grain filling. Another study, using high concentration of ozone (daytime mean 71ppb, in compare with 36ppb in the control), reported that grain chalkiness was increased by abnormally in starch accumulation, and partly attributed to the repressed expression of starch synthase IIIa involved in amylopectin side-chain elongation (Sawada et al., 2016).

Besides the sink size, grain filling and starch synthase, variations among the grains located at different positions within a rice panicle could play a role in grain chalkiness under elevated O_3_, as studies reported that grain quality varies by their position in a panicle (Chen et al., 2016; Zhang, 2021).

Elevated O_3_ usually negatively affect the grain filling and grain growth, and if the concentration is high enough, the spikelet number per panicle could also be reduced (Shao et al., 2020). Although the effect was not statistically significant, we have found that EDU spray did have a trend to increase the spikelet number per panicle against the surface ozone (Zhang et al., 2021), which suggest that EDU spray could alter the occurrence of grains with different maturity through the assimilate allocation among different spikelets. EDU could regulate the activity of superoxide dismutase, catalase and other antioxidant enzymes in rice (Pandey et al., 2015), protecting grain yield against surface O_3_. Mechanisms for the reduced grain chalkiness in rice by EDU could also be explained by the metabolic regulations, whose details remains to be revealed. Studies on the possibility of developing adaptations to raising levels of ozone by adjusting ethylenediurea application should also be conducted in future.

## Conclusion

The effects of ethylenediurea on grain chalkiness in rice cultivars are important, particularly in Asian countries like China and India, where rice is the premier staple crop consumed as grains and the concentrations of surface ozone are rising rapidly. The increased grain chalkiness will deteriorate quality of the grains and lower the price of grains on the market. The rise of surface ozone level will therefore exert greater damages to the food supply and household economy than the loss of grain mass harvested. The genetic variability in the effects of ethylenediurea on the grain chalkiness should offer a possibility of better understanding the mechanisms of ozone damages to grain filling process as well as that of screening for tolerant varieties as an adaptive measure to the rising ozone levels.

## Data availability statement

The raw data supporting the conclusions of this article will be made available by the authors, without undue reservation.

## Author contributions

GZ: conceptualization, methodology, investigation, data curation, formal analysis, funding acquisition, and writing – original draft. HR: investigation and formal analysis. KK: statistical analyses, visualization, and writing – review and editing. RC, QH, XP, YH, HW, and BS: investigation. ZZ: conceptualization and resources. ZF: conceptualization, project administration, and funding and resources acquisition. All authors contributed to the article and approved the submitted version.
