# Supplementary material for: Ethylenediurea reduces grain chalkiness in hybrid rice cultivars under ambient levels of surface ozone in China
Source: Front Plant Sci. 2022 Sep 1;13:983576. doi: 10.3389/fpls.2022.983576 (PMC9479492; doi:10.3389/fpls.2022.983576)
Supplement: Supplementary file 1 [file Table_1.docx]

**Supplementary material:** Visual inspection of chalky grains in rice.

A perfect grain in rice is translucent, whereas chalky areas in the chalky grains prevent transmission of scattered light. Chalky grains are categorized according to the position of the chalky area within the grain. The figure below, which is cited from the published paper by Yoshioka et al. in 2007 (https://doi.org/10.2135/cropsci2006.10.0631sc) shows the different types of chalky grain, including perfect rice (PR), white-based rice (WBSR), white-back rice (WBCR), white-back and -based rice (WBBR), white-belly rice (WBR), white-core rice (WCR), and milky-white rice (MWR).


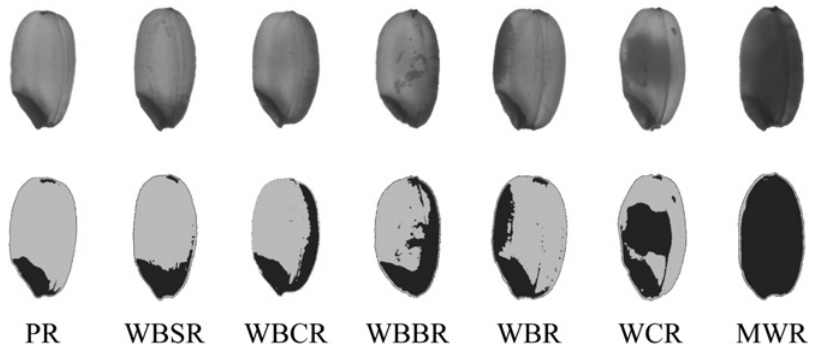


Figure S1. Grayscale images (upper row) and binary images (lower row) of rice grains: perfect rice (PR), white-based rice (WBSR), white-back rice (WBCR), white-back and -based rice (WBBR), white-belly rice (WBR), white-core rice (WCR), and milky-white rice (MWR). Since the grains were illuminated from behind, the chalky parts of the grains appear darker than normal parts. Cited from Yoshioka et al. (2007, https://doi.org/10.2135/cropsci2006.10.0631sc)
